# CHA2DS2 VASc score and brachial artery flow-mediated dilation as predictors for no-reflow phenomenon in patients with ST-segment elevation myocardial infarction undergoing primary percutaneous coronary intervention

**DOI:** 10.1186/s43044-022-00249-x

**Published:** 2022-02-23

**Authors:** Mohamed Ismail Rashed, Mohamed Ayman Saleh, Ehab Mohamed Elfekky, Ahmed Mohamed Elmahmoudy

**Affiliations:** Department of Cardiology, Ain Shams University, 38 Ramsis Street, El Abbaseya, Cairo, Egypt

**Keywords:** CHA2DS2 VASc score, Endothelial dysfunction, Flow-mediated dilation, No-reflow, ST-segment elevation myocardial infarction

## Abstract

**Background:**

Following primary percutaneous coronary intervention (PCI), no-reflow is associated with a high rate of long-term unfavorable clinical outcomes. Despite the importance of early no-reflow prediction in cardiovascular medicine, noninvasive assessment is lacking. This study aimed to evaluate the preprocedural CHA2DS2 VASc score and the brachial artery flow-mediated dilation percentage (FMD%) as predictors of the no-reflow phenomenon in patients with acute ST-segment elevation myocardial infarction (STEMI) undergoing primary PCI.

**Results:**

This study included 150 patients who presented with acute STEMI, underwent primary PCI, and were divided into two groups according to the flow result, reflow group and a no-reflow group. The CHA2DS2 VASc score was calculated and evaluation of endothelial function by measuring the brachial artery FDM% was done for each patient before the procedure. There were 39 (26%) patients in the no-reflow group and 111 (74%) in the reflow group. The no-reflow patients were older and had significantly higher body mass index (BMI), higher frequency of diabetes mellitus, hypertension, history of heart failure, dyslipidemia, Killip class IV on admission, thrombus grade V, multiple affected vessels, conventional stenting, and multiple stents placement, longer ischemic times, higher CHA2DS VASc score, and lower brachial artery FMD% (*p*-values of < 0.05 for all). Moreover, there was a significant negative correlation between CHA2DS VAS score and preprocedural FMD%, with the higher the score indicating lower FMD among cases (*p*-value = 0.000).

**Conclusions:**

Preprocedural CHA2DS2 VASc score and the brachial artery FMD can be used as predictors for the no-reflow phenomenon in patients with STEMI, undergoing primary PCI.

## Background

In patients having ST-segment elevation myocardial infarction (STEMI), successful revascularization of the infarct-related vessel by primary percutaneous coronary intervention (PCI) doesn't always recover the micro-vascular tissue perfusion to its normal state; "No-reflow" is how this is actually described [1]. No-reflow constituted widely from 11 to 41% of all the patients presented with acute STEMI [9]. Following primary PCI, no-reflow is related to various major adverse cardiac events [2]. So, no reflow is better to be early predicted and prevented to change the prognosis of primary PCI. The CHA2DS2 VASc score is a clinical predictor of thromboembolic events in patients with non-valvular atrial fibrillation. It incorporates traditional risk variables linked to atherosclerosis and consequent endothelial dysfunction, one of the underlying reasons for no-reflow [3]. The flow-mediated dilation (FMD) of the brachial artery is one of the noninvasive endothelial function tests that reflect the endothelial health of the entire arterial system, including the coronary arteries. [4].

## Methods

This is an observational cross-sectional case–control study in which we enrolled 150 patients who presented to a tertiary care university hospital with acute STEMI among period from January 2020 till June 2021. Patients were classified into two main groups according to the flow result after PCI, no-reflow group and a reflow group, the later was used as a control group. Patients treated with fibrinolytic therapy, patients whose coronary anatomy was not eligible to perform PCI, patients with known chronic inflammatory diseases, and those who died during the PCI procedure were ruled out of the study. Study design was revised and approved by the local ethical committee (FMASU MD 396/2019) following declaration of Helsinki last updated 2008 and an informed written consent was obtained from each patient. All participants were subjected to detailed medical history and thorough clinical examination.

CHA2DS2 VASc score was calculated for all participants by giving 1 point to previously established risk factors (congestive heart failure or left ventricular ejection fraction (LVEF) ≤ 40%, hypertension, diabetes mellitus, age 65–74, vascular disease and sex (female category)), age ≥ 75, and previous stroke or transient ischemic attack score 2 points [5].

The brachial artery duplex was performed immediately before coronary angiography during patient preparation in the cath lab using a General Electric (GE) vivid-e machine using 7.5 MHz linear array transducer with clear anterior and posterior intimal surfaces to obtain a baseline resting longitudinal image of the brachial artery using B mode. After that, a sphygmomanometric cuff was placed above the antecubital fossa, and the cuff was inflated to 50 mm Hg above systolic pressure for a standardized time (3 min), followed by cuff deflation. The post dilatation image of the artery was recorded 30 s post deflation to detect the maximal reactive hyperemia. The FMD% = (post dilatation diameter of reactive hyperemia-baseline diameter) / baseline diameter multiplied by 100 [6]. The interventional cardiologist was blinded to the results of the brachial artery FMD.

No reflow was angiographically defined: as Thrombolysis in Myocardial Infarction (TIMI) flow < III or TIMI III flow but with myocardial blush grade (MBG) 0 to 1 despite the mechanical reopening of the culprit vessel without dissection or thromboembolic complications [7]. Glycoprotein IIb/IIIa inhibitors, intracoronary vasodilators, and thrombus aspiration devices were used when available only as a bailout therapy. The flow result was evaluated after stent placement by two experienced cardiologists and reached a consensus on the findings.

### Statistical analysis

Data was gathered, coded, and entered into IBM SPSS version 23 (Statistical Package for Social Science). Mean and standard deviation described parametric numerical data, while Median and Interquartile range (IQR) for non-parametric numerical data. Non-numerical variables were presented as frequency and percentage. Analyses of qualitative variables were performed by chi-square and fisher exact tests. Parametric variables were analyzed by independent t test, and nonparametric variables were analyzed by Mann–Whitney U and Kruskal–Wallis tests. Univariate and multivariate regression analyses were applied to determine the independent predictors of no-reflow. Using receiver operating characteristic curves (ROC), we determined the prediction utility of the CHA2DS2-VASc score and brachial artery FMD for no-reflow. The setting for the confidence interval was 95% and the margin of error accepted was set to 5%, so the *p*-value was considered significant if *p* < 0.05.

## Results

### Baseline clinical characteristics and angiography results

Thirty nine patients developed no-reflow complication (26%) after 1ry PCI. Patients in the no-reflow group were significantly older than those in the reflow group (*p*-value = 0.039). The mean BMI was significantly higher in the no-reflow group than in the reflow group (*p*-value = 0.031). Moreover, the frequencies of diabetes mellitus, hypertension, history of heart failure, and dyslipidemia were significantly higher in the no-reflow group compared to those in the reflow group (*p*-values = 0.001, 0.000, 0.010, and 0.000 respectively) (Table 1).

**Table 1:** Demographics and risk factors of the studied patients

		Flow result		*P*-value
		Reflow	No reflow	
Age	Mean ± SD	53.92 ± 9.25	57.56 ± 9.86	0.039
	Range	33–77	36–76	
BMI kg/m^2^	Mean ± SD	27.75 ± 2.56	28.82 ± 2.88	0.031
	Range	23–35	24–35	
Diabetes mellitus	No	80 (72.1%)	16 (41.0%)	0.001
	Yes	31 (27.9%)	23 (59.0%)	
Hypertension	No	80 (72.1%)	9 (23.1%)	0.000
	Yes	31 (27.9%)	30 (76.9%)	
History of heart failure	No	108 (97.3%)	33 (84.6%)	0.010
	Yes	3 (2.7%)	6 (15.4%)	
Dyslipidemia	No	87 (78.4%)	9 (23.1%)	0.000
	Yes	24 (21.6%)	30 (76.9%)	

BMI, body mass index

The frequency of Killip class IV was significantly higher in the no-reflow group compared to those in the reflow group (20.5% vs. 2.7%). The medians of pain to door and door to wire times were significantly higher in the no reflow group compared to those in the reflow group (*p*-values = 0.000, and 0.020 respectively) (Table 2).

**Table 2:** Ischemic times of the studied patients

		Flow result		*P*-value
		Reflow	No reflow	
Pain to door time (h)	Median (IQR)	6 (3–10)	10 (8–12)	0.000
	Range	1–24	6–35	
Door to wire time (min)	Mean ± SD	21.22 ± 2.80	22.44 ± 2.78	0.020
	Range	20–35	20–30	

No-reflow and reflow groups did not statistically differ in gender (*p*-value = 0.772), smoking status (64.1% vs. 69.4%, *p*-value = 0.544), family history of coronary artery disease (10.3% vs. 5.4%, *p*-value = 0.296), peripheral arterial diseases (5.1% vs. 4.5%, *p*-value = 1.000), and chronic kidney diseases (5.1% vs. 1.8%, *p*-value = 0.277). Also, both groups didn't statistically differ in culprit type (*p*-value = 0.054), stent type, and post stenting dilatation (*p*-value = 0.365, 0.277 respectively).

The incidence of no-reflow was 15% among the direct stenting group and the conventional stenting group was 33% with a significant relation between PTCA and no reflow (*p*-value = 0.000). In addition, the patients in the no-reflow group had > 1 affected vessel and underwent > 1 stent placement compared to those in the reflow group (66.7% vs. 37.8% and *p*-value = 0.002, 28.2% vs.10.8% and *p*-value = 0.010 respectively). The frequency of thrombus grade V was higher in the no reflow group compared to those in the reflow group (35.9% vs. 18.9%) with a significant relation between thrombus grade and no reflow (*p*-value = 0.006).

### Relation between the FMD percentage, CHA2DS VASc score, and the angiographic results

The median of FMD% was significantly higher in patients with TIMI III flow and MBG ≥ II compared to those with TIMI < III and MBG < II (*p*-value = 0.000, and 0.027 respectively) (Table 3).

**Table 3:** Relation between FMD% and the TIMI flow, the MBG

		FMD%		*P*-value
		Median (IQR)	Range	
TIMI flow	I	6 (4–20)	1–41	0.000
	II	5 (2–10)	0–41	
	III	26 (20–32)	0–58	
MBG	I	2 (0–16)	0–16	0.027
	II	26 (20–36)	0–45	
	III	26 (22–32)	6–58	

TIMI, Thrombolysis in Myocardial Infarction; MBG, myocardial blush grade

The median CHA2DS VASc score was significantly higher in patients with TIMI flow < III and MBG < II compared to those with TIMI III and MBG ≥ II (*p*-value = 0.000, and 0.002 respectively) (Table 4).

**Table 4:** Relation between the CHA2DS VASc score and the TIMI flow, the MBG

		CHA2DS VAS score		*P*-value
		Median (IQR)	Range	
TIMI flow	I	3 (2–5)	1–7	0.000
	II	4 (3–5)	1–6	
	III	2 (1–3)	1–5	
MBG	I	4 (2–5)	2–5	0.002
	II	2 (1–3)	1–4	
	III	1 (1–2)	1–3	

TIMI, Thrombolysis in Myocardial Infarction; MBG: myocardial blush grade

### Multiple regression analysis to study independent factors for no-reflow

After performing regression univariate analysis of factors with significant effect as regards the incidence of no-reflow, the multivariate regression analysis of different independent factors showed significant value only as regards FMD% ≤ 11% (*p* = 0.000) (odds ratio 68.250) (Table 5).

**Table 5:** Multiple regression of the independent predictors for no-reflow

	Uni-variety				Multi-variety			
	*P*-value	Odds ratio (OR)	95% C.I. for OR		*P*-value	Odds ratio (OR)	95% CI for OR	
			Lower	Upper			Lower	Upper
Age (> 59 years)	0.008	2.816	1.317	6.022	–	–	–	–
BMI (> 30)	0.005	3.667	1.481	9.075	–	–	–	–
Diabetes Mellitus	0.001	3.710	1.733	7.940	–	–	–	–
Hb A1c (> 8.3)	0.003	6.286	1.890	20.901	–	–	–	–
Hypertension	0.000	8.602	3.667	20.177	–	–	–	–
Dyslipidemia	0.000	12.083	5.056	28.878	–	–	–	–
History of heart failure	0.011	6.545	1.551	27.618	–	–	–	–
Killip classify	0.003	1.891	1.243	2.879	–	–	–	–
Diseased vessels num	0.002	3.286	1.524	7.085	–	–	–	–
Thrombus grade	0.001	1.699	1.243	2.320	–	–	–	–
PTCA	0.000	3.063	1.677	5.595	–	–	–	–
Stents number	0.012	3.241	1.292	8.128	–	–	–	–
Stent length (> 33 mm)	0.008	2.758	1.303	5.836	–	–	–	–
CHA2DS VASc > 3	0.000	18.508	7.075	48.412	–	–	–	–
FMD% ≤ 11%	0.000	70.667	22.022	226.764	0.000	68.250	11.371	409.656

BMI, body mass index; Hb A1c, glycated haemoglobin; PTCA, percutaneous coronary angioplasty; FMD, flow-mediated dilation

### A ROC curve analysis of CHA2DS2 VASc score and FMD%

**Fig. 1:**
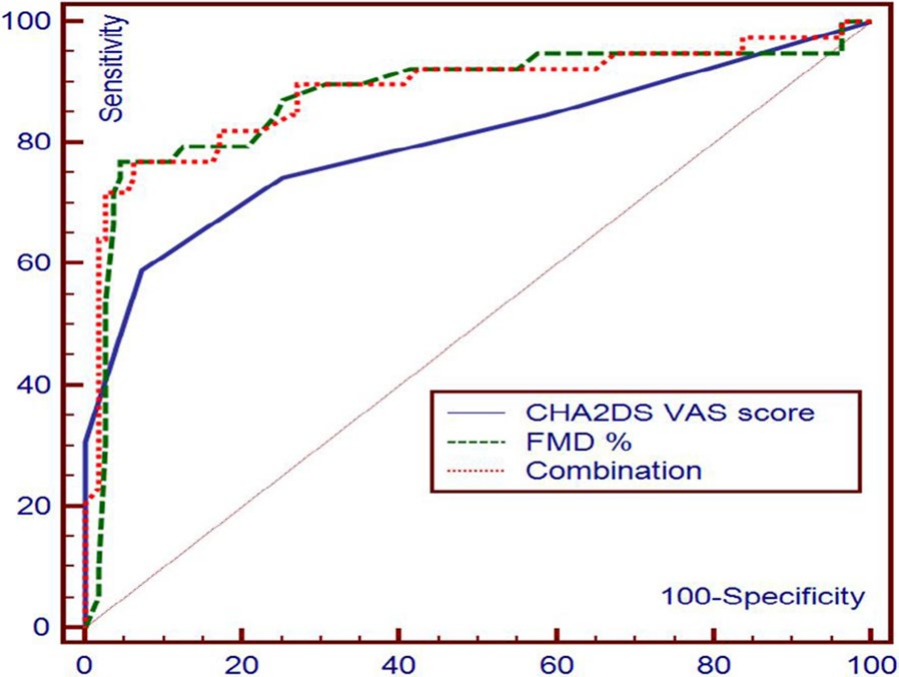
ROC curve for the CHA2DS VASc score and the FMD percentage

A ROC curve analysis was done and showed that the best cut-off value of the CHA2DS2 VASc score to predict no-reflow was > 3 with a sensitivity of 58.97% and a specificity of 92.79% (area under ROC curve = 0.800). A ROC curve comparison analysis also showed the best cut-off value of FMD% to predict no-reflow was ≤ 11% with a sensitivity of 76.92% and a specificity of 95.5% (area under ROC curve = 0.878). And combining both cut-off values can predict no-reflow with a sensitivity of 76.92% and a specificity of 93.69% (area under ROC curve = 0.885) (Fig. 1) (Table 6).

**Table 6:** The cut off values for prediction of no reflow using the CHA2DS VASc score and the brachial artery FMD%

Parameter	Area under ROC	Cut of point	Sensitivity (%)	Specificity (%)	Positive predictive value (%)	Negative predictive value (%)
CHA2DS VAS score	0.800	> 3	58.97	92.79	74.2	86.6
FMD%	0.878	≤ 11	76.92	95.5	85.7	92.2
Combination	0.885	–	76.92	93.69	81.1	92.0

ROC, receiver operating characteristic curve

### Correlation between the FMD percentage and the CHA2DS VAS score

**Fig. 2:**
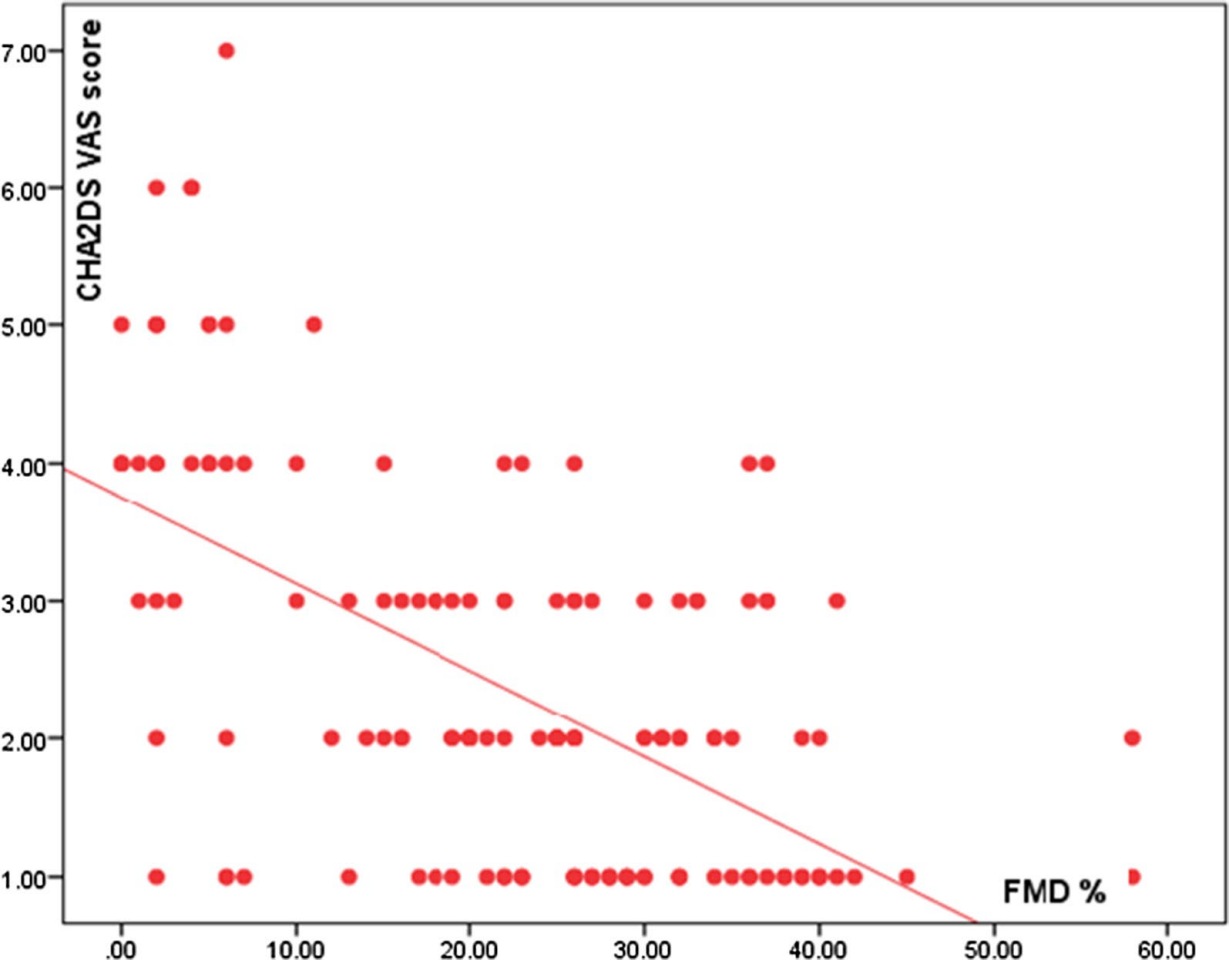
Correlation between the FMD percentage and the CHA2DS VAS score

In the study, there was a significant negative correlation between CHA2DS VAS score and preprocedural FMD%, with the higher the score indicating lower FMD among cases (*p*-value = 0.000) (Fig. 2).

### Description of LV systolic function and inpatient major adverse cardiovascular events (MACE) among study cases

74% of the studied population (111 patients) had LV dysfunction (an EF < 50%) and 26% (39 patients) had an EF of 50% or more. No significant relation was found between the patients' CHA2DS VASc scores, brachial artery FMD, and the incidence of major adverse cardiovascular events during the hospital stay (*p*-value = 0.164, and 0.723, respectively).

## Discussion

Our study evaluated the preprocedural CHA2DS2-VASc score and the brachial arteries FMD as predictors for the no reflow phenomenon in patients with STEMI undergoing primary PCI. The rate of no-reflow following primary PCI in our study was 26%, which was in agreement with previously described no-reflow rates between 11 and 41% with variation between different studies according to the patient characteristics, angiographic factors, and management methods [9].

The current study evaluated the risk factors with a statistically significant effect on the incidence of no-reflow using univariate regression analysis. Those factors included age > 59 years, BMI > 30 m^2^/kg, diabetes mellitus, glycated haemoglobin (HbA1c) among diabetic patients (> 8.3%), hypertension, history of heart failure, dyslipidemia, Killip class on admission, number of affected vessels, thrombus grade, PTCA, stents number, stent length > 33 mm. Yang et al. [8] revealed that low systolic blood pressure on admission < 100 mmHg, and pre PCI high thrombus score were risk factors for no-reflow as confirmed by our study findings. In another study, advanced age, prolonged pain to door time, and Killip class IV on admission were demonstrated to be independent predictors for no-reflow [9]. Moreover, Durante et al. [10] found that hypertension, dyslipidemia, and larger ischemic regions were independent risk factors for no-reflow. Dean et al. [11] also discovered an association between diabetes mellitus and micro-vascular dysfunction, one of the underlying processes of no-reflow. Also, 443 patients with acute STEMI and underwent primary PCI were studied in another study that found a significant statistical relation between the HbA1c level and one year 1ry adverse cardiovascular complications, including mortality, recurrent ischemia, and stroke [12]. Our studied patients with longer pain-to-door time had a significantly greater thrombus burden and an increase in no-reflow rates than patients with short reperfusion times (*p*-value = 0.000) and this was confirmed by Sabin et al. and his colleagues [13], who showed that patients with a long time before reperfusion (> 6 h) had a considerably higher thrombus burden and a 1.3 fold greater no-reflow rate than those with a short reperfusion interval. The incidence of angiographic no-reflow among our studied population was 15% among the direct stenting group and the conventional stenting group was 33%. This ratio between both groups is similar to the ratio of Antoniucci et al. [14] showing that the incidence of angiographic no-reflow was 12% in the conventional stenting group and 5.5% in the direct stenting group (*p*-value = 0.040).

The current study's findings confirmed the clinical significance of the CHA2DS2 VASc score in predicting no-reflow after primary PCI where the CHA2DS VASc score was significantly higher in patients with < TIMI III flow (*p*-value = 0.000) and patients with MBG < II (*p*-value = 0.002) compared to those with TIMI III and MBG ≥ II. These findings were consistent with those of Mirbolouk et al. [15], who discovered that the CHA2DS2VASc score was an important predictor of no-reflow in 398 patients with acute STEMI (*p*-value = 0.001). Significantly, the CHA2DS2 VASc score was associated with suboptimal revascularization and short-term adverse cardiovascular outcomes after primary PCI in patients with acute STEMI, which may indicate that the score effectively predicts the outcomes in patients by predicting no-reflow [16]. So, high-risk patients for no reflow could be identified with simpler risk scores, more effective medications and follow up of patients may be given.

The median of FMD% in the studied population was higher in patients with TIMI III Flow (*p*-value = 0.000) and in patients with MBG ≥ II (*p*-value = 0.027) than in those with TIMI < III flow and MBG < II adding a value in addition to the CHA2DS2 VASc score to identify high-risk patients for no-reflow and follow up them. In addition, the multivariate regression analysis of different independent factors increasing no-reflow risk showed significant value only for FMD% ≤ 11% (*p*-value = 0.000) (odds ratio = 68.250). A limited number of studies have tried to identify the relation between endothelial dysfunction, and suboptimal reperfusion, and short-term adverse cardiac events after primary PCI. In a recent study, the endothelial function was assessed for patients with acute STEMI using brachial artery FMD during a hospital stay after being revascularized by primary PCI and a strong correlation was found between the FMD and the MBG among the studied population (*p* = 0.029) [17]. However, drugs with a well-established effect on improving endothelial dysfunction such as nitrates and statins could have influenced the results of that study, which was avoided in our study by doing the FMD test during patient preparation in the cath lab before the procedure. Neunteufl et al. [18] also showed that endothelial dysfunction using FMD is related to cardiovascular complications in patients without known IHD but have chest pain and have undergone coronary angiography. In addition, Karatzis et al. [19] found that FMD is an independent indicator of subsequent cardiovascular events in patients who survive non-STEMI admission [19]. Furthermore, intravascular ultrasonography has recently confirmed the link between a lower FMD value and a larger necrotic core in coronary plaque in both culprit and non-culprit arteries [20]. In 26 individuals without any coronary plaques and with only sluggish coronary flow, Ari et al. [21] found a statistically significant negative correlation between FMD and TIMI frame count (*p*-value = 0.004). This study differs from ours in terms of the group population and the patient preparation. However, the independent relationship between nitric oxide synthase inhibitor and brachial artery FMD, as well as the association between impaired brachial artery FMD and higher TIMI frame count, validated the significance of nitric oxide and its production inhibitors in endothelial dysfunction in coronary vessels. The results of Ari et al. [21] enforced our work theory that there is an obvious correlation between endothelial dysfunction and slow coronary flow even in angiographically normal coronary arteries. Thus, before and after the primary PCI, a clinical assessment of the brachial artery FMD gives valuable prognostic information in the long-term care of patients at high risk for no-reflow.

There was a significant negative correlation between the CHA2DS VAS score and preprocedural FMD% in our study, with the higher the score indicating lower FMD among cases (*p*-value = 0.000). Endothelial dysfunction is an important risk factor in most cardiovascular diseases, and it is associated with advanced age, hypertension, diabetes mellitus, and vascular disease and these factors are part of the CHA2DS2-VASc score [22]. So, FMD is expected to be impaired in a patient with a higher CHA2DS VAS score as suggested from the current study.

The current study didn't find a significant relationship between the CHA2DS VASc score or FMD% and the incidence of inpatient MACE (*p*-value = 0.164, 0.723 respectively). These findings differed from those of Guazzi et al. [23], who discovered a significant correlation (*p*-value = 0.01) between the values of brachial artery FMD in 197 patients who presented with myocardial infarction and the incidence of MACE over a period of 13.7 (± 9.5) months [23]. The discrepancy between the two trials could be attributable to the Guazzi et al. study having a longer follow-up period and a larger number of participants. However, Frick et al. [24] discovered a non-significant correlation (*p*-value = 0.79) between brachial artery FMD and MACE in 398 male patients who underwent coronary angiography due to chest pain over a 4-year follow up period. Patients admitted with acute coronary syndrome were omitted from Frick's study, which could explain the disparity in results between Frick et al. [24] and Guazzi et al. [23].

### Limitations

The study is based on single-center data with a small sample size. Also, micro-vascular perfusion was not accurately assessed by nuclear imaging or better by cardiac magnetic resonance imaging. In addition, the short follow-up period made the MACE assessment suboptimal.

## Conclusions

The study presented the value of preprocedural CHA2DS2 VASc score and the brachial artery FMD as predictors for the no-reflow phenomenon in STEMI patients undergoing primary PCI.

## Data Availability

The datasets used and analyzed during the current study are available from the corresponding author on reasonable request.

## Abbreviations

BMI: Body mass index
FMD%: Flow-mediated dilation percentage
GE: General electric
HbA1c: Glycated haemoglobin
IQR: Interquartile range
LVEF: Left ventricular ejection fraction
MACE: Major adverse cardiovascular events
MBG: Myocardial blush grade
PCI: Percutaneous coronary intervention
PTCA: Percutaneous Coronary Angioplasty
ROC: Receiver operating characteristic
SPSS: Statistical Package for Social Science
STEMI: ST-segment elevation myocardial infarction
TIMI: Thrombolysis in Myocardial Infarction
